# Effects of Oral Glucose-Lowering Agents on Gut Microbiota and Microbial Metabolites

**DOI:** 10.3389/fendo.2022.905171

**Published:** 2022-07-13

**Authors:** Dongmei Wang, Jieying Liu, Liyuan Zhou, Qian Zhang, Ming Li, Xinhua Xiao

**Affiliations:** ^1^ Department of Endocrinology, National Health Commission (NHC) Key Laboratory of Endocrinology, Chinese Academy of Medical Sciences, Peking Union Medical College Hospital, Peking Union Medical College, Beijing, China; ^2^ Department of Medical Research Center, Chinese Academy of Medical Sciences, Peking Union Medical College Hospital, Peking Union Medical College, Beijing, China

**Keywords:** gut microbiota, microbial metabolites, T2DM, antidiabetic drugs, SCFA

## Abstract

The current research and existing facts indicate that type 2 diabetes mellitus (T2DM) is characterized by gut microbiota dysbiosis and disturbed microbial metabolites. Oral glucose-lowering drugs are reported with pleiotropic beneficial effects, including not only a decrease in glucose level but also weight loss, antihypertension, anti-inflammation, and cardiovascular protection, but the underlying mechanisms are still not clear. Evidence can be found showing that oral glucose-lowering drugs might modify the gut microbiome and thereby alter gastrointestinal metabolites to improve host health. Although the connections among gut microbial communities, microbial metabolites, and T2DM are complex, figuring out how antidiabetic agents shape the gut microbiome is vital for optimizing the treatment, meaningful for the instruction for probiotic therapy and gut microbiota transplantation in T2DM. In this review, we focused on the literatures in gut microbiota and its metabolite profile alterations beneficial from oral antidiabetic drugs, trying to provide implications for future study in the developing field of these drugs, such as combination therapies, pre- and probiotics intervention in T2DM, and subjects with pregestational diabetes and gestational diabetes mellitus.

## Introduction

The International Diabetes Federation Diabetes Atlas 10th edition shows a continued global increase in diabetes prevalence, estimating that 537 million adults are living with diabetes worldwide, most of which is type 2 diabetes mellitus (T2DM) (1). T2DM is a metabolic disorder with multiple pathogenic factors, including genetic elements, sedentary behaviors, and overeating (2). Once without effective treatment, it might lead to a composite of microvascular or macrovascular complications, for instance chronic kidney disease, diabetic eye disease, and cardiovascular disease (CVD) (3). Differing from insulin-dependent type 1 diabetes mellitus, T2DM is closely interrelated with insulin resistance (IR) and strongly intertwined with obesity, non-alcoholic fatty liver disease, and metabolic syndrome (4). Nowadays, more than 10 types of medicines are approved by the USA Food and Drug Administration for the glycemic treatment (5). Thousands of clinical trials and basic research are proceeding worldwide for diabetes pharmacotherapy, including looking for potential intervention targets (6). In addition to the reduction in HbA_1_c, results from a vast number of clinical and experimental studies have shown the potential effects of glucose-lowering drugs, such as weight reduction, cardiovascular safety, and lipid-lowering and antihypertensive effects; however, the mechanisms behind these benefits need to be further revealed (5, 6).

Gut microbiota has become a hot topic in metabolic disorders in the past decade, including T2DM (7–9). Accumulating evidence confirms that gut microbiota has emerged as a large complex ecological community and a vital regulator of host physical condition, *via* microbial metabolites and host interactions (10, 11). Among 100 trillion of microorganisms, which is 10 times the number of human body cells, including bacteria, fungi, viruses, and protozoa, the bacterial component is characterized as the most well-investigated group (11, 12) and will be the chief spotlight of this review. There are nearly 500–1,000 species of bacteria within the gastrointestinal tract and more than 90% of the total community are Firmicutes and Bacteroidetes at the phylum level, followed by Proteobacteria, Actinobacteria, Verrucomicrobiota, Fusobacteria, Cyanobacteria, and Tenericutes (11, 12). The gut microbiota homeostasis is preserved with control of pathogenic microbe growth and protection of beneficial microbes (11, 13). The gut microbiome is considered as a modifiable “new organ” that plays a crucial role in shaping the metabolic and immunological functions of T2DM (14). Although with wide interindividual variation, once the gut microbiota composition was destroyed, an imbalanced gut microbiome community leads to an abnormal production of metabolites, lipid and carbohydrate metabolism disturbance, IR, oxidative stress, and low-grade chronic inflammatory state in T2DM (7, 8, 15–18).

Therefore, understanding how antidiabetes agents influence the gut microbiome might be of importance for optimizing T2DM treatment. Microbiota and host metabolism might deliver promising and novel constructive aspects of commonly used oral antidiabetic drugs (19). In addition, fecal microbiota transplantation (FMT) has become a promising strategy for patients with T2DM (20, 21). In this review, we focus on the literatures in gut microbiota and metabolite profile alterations beneficial from oral antidiabetic drugs in diabetes and metabolic disorder state, in both basic research and clinical studies. We aim to figure out the similarities and differences in the literatures of gut microbiota and the metabolite-related effect of oral antidiabetic drugs, in order to deliver some leads for future studies in these developing fields of these drugs and T2DM treatment.

## Gut Microbiota and Metabolites Altered in T2DM

Although the definite microbial signatures linked to T2DM have not been discovered yet, a large number of studies have found that gut microbiota dysbiosis in T2DM is highly associated with specific intestinal microbial taxa or certain enrichment of gene functional pathways (22–28). In a metagenome-wide association study from 345 Chinese individuals, T2DM-related gut flora dysbiosis was characterized by a decreased abundance in a cluster of butyrate-producing bacteria, such as *Roseburia intestinalis*, *Faecalibacterium prausnitzii*, *Clostridiales* sp. SS3/4, and *Eubacterium rectale*, and an increased abundance of opportunistic pathogens, such as *Bacteroides caccae*, *Escherichia coli*, and some *Clostridium* species (*Clostridium symbiosum*, *Clostridium bolteae*, *Clostridium hathewayi*, and *Clostridium ramosum*) (22). Another large-scale metagenome analysis study which recruited a population of 145 70-year-old European women with metagenomic profiles showed increases in the abundance of four *Lactobacillus* species (including *Lactobacillus gasseri*), *Streptococcus mutans*, and *Clostridium hathewayi* and decreases in the abundance of five *Clostridium* species (including *Clostridium beijerinckii*, *Clostridium botulinum*), *Roseburia_272*, and *Bacteroides intestinalis* in the T2DM group (23). Due to the difference in genetic inheritance, diet, and lifestyle factors, the connections among gut microbial communities, microbial metabolites, and T2DM are intricate. Despite the obvious discrepancy in metagenomic clusters between these two populations, the similar microbial functions enriched in T2DM included an increased level in lipid or glucose metabolism-related membrane transport and oxidative stress resistance and a decreased level in metabolism of vitamins and cofactors, butyrate production, and cell motility (22, 23). To recognize the core gut microbial features of T2DM, a machine learning framework totally recruited more than 9,000 people revealed that a microbiome risk score including 14 microbial features was positively associated with risk of T2DM and the future glucose increment after adjustment for traditional risk factors (such as age, sex, parental history of diabetes, body mass index, systolic blood pressure, and triglycerides) (28). In the meantime, a downward trend of butyrate-producing genus (*Roseburia* spp.) and a rising trend of chronic inflammation-associated genus (*f:lactobacillaceae*) were confirmed in this interpretable machine learning framework (28). Among a substantial body of experimental and clinical research, the genera of *Bifidobacterium*, *Akkermansia*, *Bacteroides*, *Roseburia*, and *Faecalibacterium* were inversely correlated with T2DM, while the genera of *Ruminococcus*, *Blautia*, *Lactobacillus*, and *Fusobacterium* were positively correlated with T2DM (8, 22, 23).

**Figure 1:**
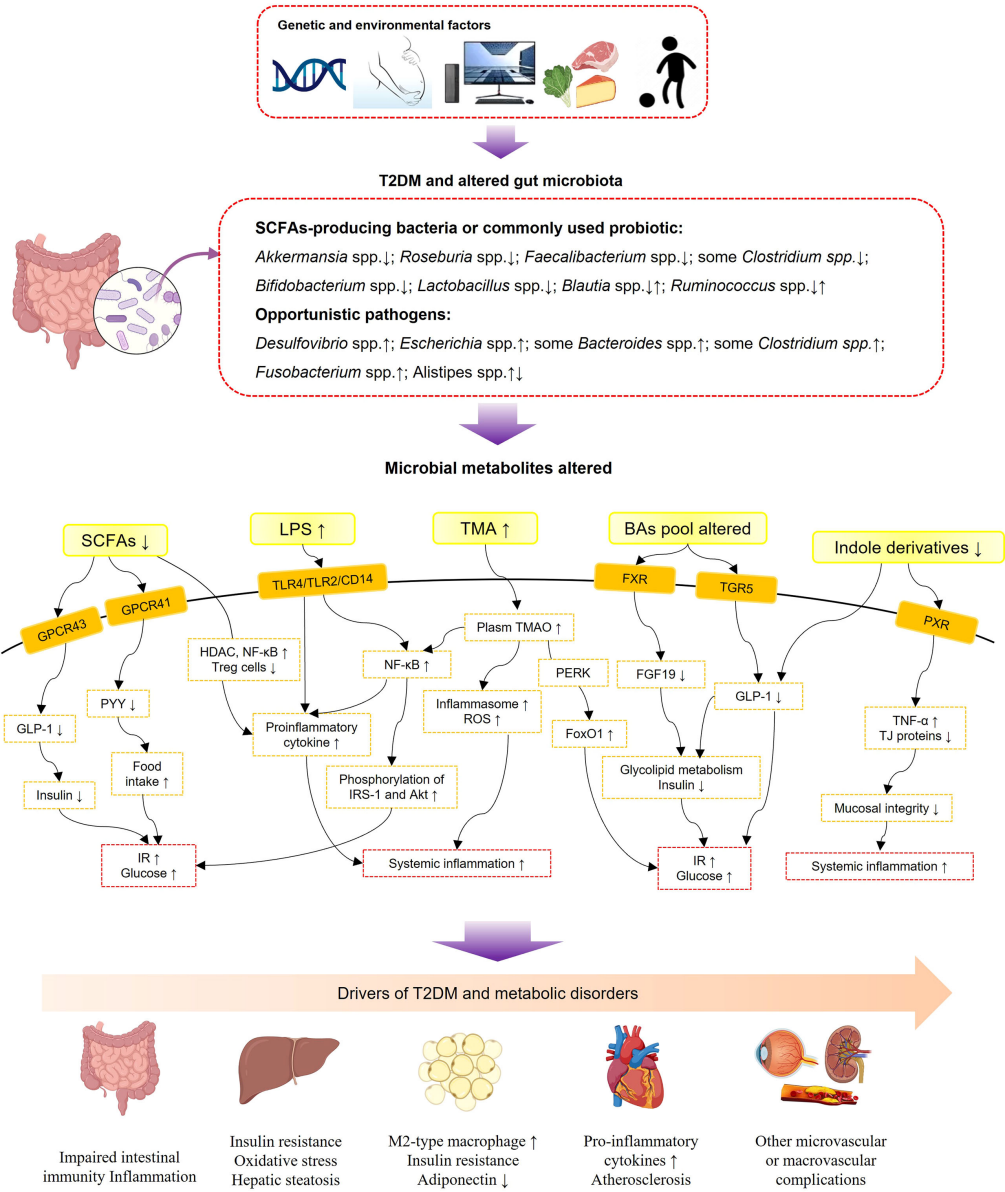
Schematic view of gut microbiota, microbial metabolites, and T2DM-associated metabolic disorders. SCFAs, short-chain fatty acids; LPS, lipopolysaccharides; TMA, trimethylamine; TMAO, trimethylamine-N-oxide; BAs, bile acids; GPCR43, G-protein-coupled receptor 43; GPCR41, G-protein-coupled receptor 41; TLR4, toll-like receptor 4; TLR2, toll-like receptor 4; CD14, cluster of differentiation 14; FXR, farnesoid X receptor; TGR5, Takeda G protein-coupled receptor 5; PXR, pregnane X receptor; GLP-1, glucagon-likepeptide-1; PYY, peptide tyrosine-tyrosine; HDAC, histone deacetylases; NF-κB, nuclear factor-kappaB; IRS-1, insulin receptor substrate-1; ROS, reactive oxygen species; PERK, protein kinase-like ER kinase; FoxO1, forkhead box-O1; FGF19, fibroblast growth factor 19; TNF-α, tumor necrosis factor alpha; TJ proteins, tight-junction proteins; IR, insulin resistance.

Although the underlying mechanism between complex gut microbiota and T2DM is still unclear, evidence has shown that a variety of metabolites derived from gut flora, including short-chain fatty acids (SCFAs), glycolipid lipopolysaccharides (LPS), bile acids (BAs), trimethylamine-N-oxide (TMAO), indole derivatives, amino acids, vitamins, and one-carbon metabolites, interacted with the host as signaling molecules and were further involved in the pathophysiological process of metabolic diseases (29–40) (**Figure 1**). SCFAs (including butyrate, acetate, and propionate) are the major microbial metabolites produced by dietary fiber fermentation within the intestinal lumen (41). SCFAs were found reduced in T2DM in both clinical and experimental research (42–45). By activation of specific G protein-coupled receptor 41 and 43 (GPR41 and GPR43), SCFAs could stimulate the secretion of peptide tyrosine-tyrosine (PYY) and glucagon-like peptide-1 (GLP-1) from intestinal enteroendocrine L cells (39, 46). PPY is an important neuroendocrine hormone, regulating food intake and energy balance; reduced secretion of GLP-1 in T2DM leads to a reduction of insulin and thus impaired glucose and energy metabolism (47). Besides, SCFAs have been identified as vital mediators in maintaining intestinal immunity and systemic inflammation through upregulating anti-inflammatory regulatory T cells, inhibition of histone deacetylase, and further inhibition of inflammatory signaling pathways and proinflammatory cytokines, such as nuclear factor-kappaB (NF-κb) and tumor necrosis factor alpha (TNF-α) (37, 48).

LPSs, the main compounds of gram-negative bacterial membranes, are known as potent stimulators of inflammation (49). Evidence shows that T2DM subjects possess a high enrichment of gram-negative bacteria, particularly those belonging to Proteobacteria at the phylum level (50). Notably, the Bacteroidetes phylum also belongs to a large part of gram-negative bacteria, but a decreased abundance of Bacteroidetes was found in obesity and diabetes conditions (24, 51–54). This contradiction might be explained by the fact that the LPS produced by the Bacteroidetes phylum has a lower endotoxic activity than other gram-negative bacteria such as the Proteobacteria phylum (55). Subsequently, a high concentration of LPS produced within the gut (metabolic endotoxemia) might lead to chronic low-grade inflammation in diabetic subjects through upregulating inflammatory signaling pathways and proinflammatory cytokine secretion (56, 57). LPSs produced by gut bacteria might damage the intestinal barrier leading to a “leaky gut” syndrome, for instance, a weakened tight junction and reduced gut secretory immunoglobulin A (58). Besides, LPSs have been confirmed to result in IR due to increased IRS-1 and Akt phosphorylation (59) (**Figure 1**).

Originally synthesized from cholesterol in the liver, BAs were revealed to have a reciprocal interaction with gut microbiota *via* the gut-to-liver axis (40). Primary BAs are converted into secondary BAs by gut microbiota (40). BAs are important signaling mediators regulating energy metabolism and systematic inflammation *via* the nuclear farnesoid X receptor (FXR) and Takeda G protein-coupled receptor 5 (TGR5) (40). In subjects with diabetes and metabolic diseases, BAs’ pool composition altered (60). The altered proportion of FXR antagonistic BAs leads to an altered expression of fibroblast growth factor 19 (FGF19), which were both vital molecules for BAs and glycolipid metabolism (60). Activation of TGR5 by secondary BAs stimulates GLP-1 secretion from L cells to increase insulin secretion and glucose tolerance (61). Evidence shows that modifications of the BA pool presented a beneficial effect in bariatric surgery and antidiabetic treatment (62–64).

TMAO is predominantly generated from dietary choline, which is transformed to trimethylamine in the gut and then oxidized in the liver (31). Elevated plasma concentrations of TMAO were reported positively related with metabolic dysfunction, such as insulin resistance, CVDs, and T2DM (31, 34, 65), and various bacteria (such as *Clostridium hathewayi*, *Escherichia fergusonii*, *Providencia alcalifaciens*, and *Providencia rustigianii*) have been recognized as contributing to the production of TMAO (66). TMAO was found to play a proinflammatory role by activating the nucleotide-binding oligomerization domain-like receptor family pyrin domain-containing 3 inflammasome, accelerating reactive oxygen species generation and various proinflammatory cytokines (67). In addition, evidence in experimental research shows that TMAO promoted metabolic dysfunction by directly binding and activating protein kinase-like ER kinase, a key sensor of intracellular stress, and then enhanced transcription activity of forkhead box-O1 in the liver (31).

Indole derivatives are produced from tryptophan by the gut microflora (33). In the recent years, indole derivatives have exhibited anti-inflammatory and antidiabetic effects (68). Evidence shows that indole derivatives were able to stimulate the secretion of GLP-1 from L cells (32). Various indole derivatives have been synthesized to investigate their bioactivities and biological functions (68). Microbe-specific indoles, such as indole 3-propionic acid, were found to regulate mucosal integrity through activating the xenobiotic sensor, pregnane X receptor, to downregulate enterocyte TNF-α expression and upregulate junctional protein expression (36). In addition to the abovementioned metabolites, vitamins and cofactors produced by probiotics, such as *Bifidobacterium* and *Lactobacillus*, yield greater health benefits on patients with T2DM and metabolic diseases (69). Amino acids synthesized by the gastrointestinal microbiota were also vital factors to energy metabolism and glucose homeostasis (70). For instance, *Prevotella copri* and *Bacteroides vulgatus* were discovered as the main species mediating the association between biosynthesis of branched-chain amino acids (BCAAs) and IR, and *Prevotella copri* could induce IR, aggravate glucose intolerance, and increase circulating BCAAs levels (70).

Overall, a vast body of human studies and plentiful animal studies have suggested that T2DM was characterized by gut microbiota dysbiosis and alterations of gut microbiota-derived metabolites, which are important contributors to the pathological injury of T2DM.

## The Effects of Oral Antidiabetic Drugs on Gut Microbiota and Microbial Metabolites

### Metformin

Metformin can alleviate patients’ hyperglycemia mainly by significant suppression of glucose production in the liver (71). Activation of the master cellular energy sensor AMP-activated protein kinase (AMPK) is well documented in the mechanism of metformin but may not interpret for its complex beneficial effects (72–75). In fact, metformin was found to modify the intestinal flora community in T2DM in a vast body of clinical research and experimental animal studies (76–80) (summarized in **Table 1**).

**Table 1:** Clinical research exploring the effects of oral anti-diabetic drugs on gut microbiota in T2DM.

Anti-diabetic drugs	Subjects	Key results
Metformin (77)	784 subjects from Denmark, Switzerland and China	*Escherichia* spp.↑ *Lactobacillus* spp. ↓ Functional enrichment: SCFAs producing↑, virulence factors and gas metabolism genes↑ intestinal lipid absorption↓ LPS triggered local inflammation↓
Metformin (78)	450 subjects	Simpson’s diversity index↑ *Blautia* spp. and *Faecalibacterium* spp.↑ *Alistipes* spp., *Oscillibacter* spp., and *Bacteroides* spp.↓
Metformin (79)	40 treatment-naive T2DM	Firmicutes, *Escherichia coli, Bifidobacterium adolescentis, Akkermansia muciniphila*↑ SCFA-producing genus↑ Fecal SCFAs and plasma bile acid concentrations↑
Metformin (45)	121 subjects	*Escherichia coli* and *Ruminococcus torques*↑; *Intestinibacter bartlettii*↓ Fecal SCFAs increased at 6 mouths
Metformin (81)	23 T2DM patients	Enterobacteriaceae↑
Metformin (76)	22 newly diagnosed T2DM	*Bacteroides fragilis*↓ bile acid glycoursodeoxycholic acid↑
Metformin (82)	60 adults with a BMI ≥ 25 kg/m^2^	*Bacteroides caccae, Lachnospiraceae bacterium*↑*Bacteroides uniformis*↓ butyrate↑zonulin↓microbial butyrate-producing pathways↑
Metformin (83)	14 males with T2DM	Firmicutes↓ GLP-1, lithocholic and deoxycholic acids↑ primary bile acid↓
Metformin (84)	112 subjects	*Akkermansia muciniphila*, *Prevotella*, *Butyrivibrio, Bifidobacterium bifidum, Megasphaera*↑ *Clostridiaceae 02d06*↓
Metformin (85)	130 T2DM subjects	*Spirochaete, Turicibacter, and Fusobacterium*↑ Taurine and hypotaurine metabolism↑
Metformin (86)	30 T2DM subjects	*Bifidobacterium*
Dapagliflozin (87)	24 subjects	No significant effect on microbial composition
Empagliflozin (88)	67 T2DM with risk factors for CVD	SCHA-producing bacteria↑ Several harmful bacteria including Escherichia-Shigella, Bilophila, and Hungatella↓
Sitagliptin (89)	51 subjects	No significant effect on microbial composition
Sitagliptin (90)	57 T2DM subjects	Fecal chenodeoxycholic acid, cholic acid and ursodeoxycholic acid ↑
Vildagliptin (91)	30 T2DM subjects	*Pseudomona*s, *Klebsiella*, *Blautia, Faecalibacterium* and *Roseburia* levels altered
Saxagliptin (91)	30 T2DM subjects	*Megamonas* spp.↑; *Turicibacter* spp. ↓
Acarbose (62)	51 treatment-naive subjects	*Lactobacillus and Bifidobacterium*↑*Bacteroides*↓ Altered plasm BAs pool composition
Acarbose (92)	18 subjects	*Bifidobacterium*, *Eubacterium*, and *Lactobacillus*↑*Bacteroides*↓
Acarbose (93)	95 subjects	*Bifidobacterium longum* and *Enterococcus faecalis*↑ Plasm LPS↓
Acarbose (91)	30 T2DM subjects	Butyricimonas level increased first and then decreased during treatment
Acarbose (94)	52 prediabetes patients	*Lactobacillus* spp. and *Dialister* spp.↑ *Butyricicoccus* spp., *Phascolarctobacterium* spp. and *Ruminococcus* spp.↓
Glipizide (62)	43 treatment-naive subjects	No effect on intestinal microbiota composition
Gliclazide (87)	17 subjects	No significant effect on microbial composition

SCFAs, short-chain fatty acids; CVD, cardiovascular disease; LPS, lipopolysaccharides; GLP-1, glucagon-likepeptide-1.

Metagenomics combined with targeted metabolomic data in a randomized, placebo-controlled, double-blind study showed that metformin strongly altered the gut microbiome and its function in individuals with treatment-naive T2DM (79). Subsequently, the authors transplanted fecal samples from three donors (treatment-naive condition compared with 4-month metformin-treated condition) into germ-free mice and observed that glucose tolerance was improved in mice that received 4-month metformin-treated fecal samples, indicating a direct beneficial effect on glucose homeostasis (79). This effect might be mediated by increased SCFA-producing bacteria and the abundance of *Akkermansia muciniphila*, enriched pathways of the metabolism of vitamins and cofactors, and metalloproteins or metal transporters (79). In line with this research, a large study aimed at disentangling metformin treatment signatures in T2DM recruited 784 subjects from Denmark, Switzerland, and China and illustrated that metformin treatment significantly increased the abundance of *Escherichia* spp. and reduced that of *Intestinibacter* spp. The functional enrichment analyses demonstrated that SCFA-producing pathways and enrichment of virulence factors and gas metabolism genes were significantly enhanced, while intestinal lipid absorption and LPS-triggered intestinal inflammation were reduced (77). A randomized clinical trial which recruited 450 T2DM subjects uncovered that metformin altered the gut microbiota composition, increased the beneficial bacteria, such as *Blautia* and *Faecalibacterium*, and inhibited potential pathogen-like microbiota, for example, *Oscillibacter*, *Alistipes*, and *Bacteroides* (78). As summarized in **Table 1**, most clinical studies revealed that microbes mediated the therapeutic effects of metformin chiefly through improvement in SCFA production, BA pool composition alteration, or reduction in LPS production.

**Figure 2:**
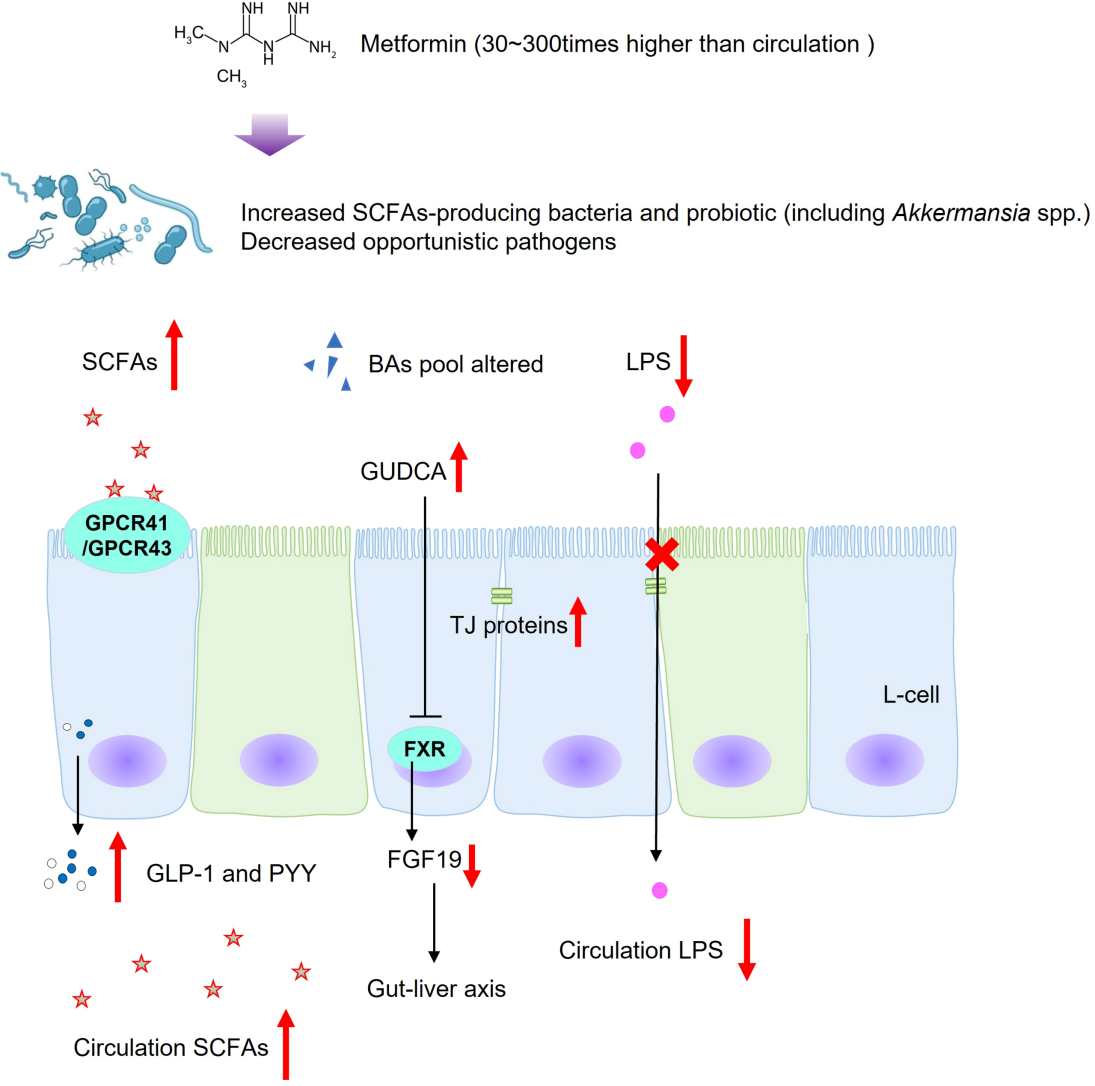
Possible regulatory mechanisms of metformin on gut microbiota and microbial metabolites in T2DM. SCFAs, short-chain fatty acids; BAs, bile acids; LPS, lipopolysaccharides; GUDCA, glycoursodeoxycholic acid; GPCR43, G-protein-coupled receptor 43; GPCR41, G-protein-coupled receptor 41; FXR, farnesoid X receptor; TGR5, Takeda G protein-coupled receptor 5; GLP-1, glucagon-likepeptide-1; PYY, peptide; tyrosine-tyrosine; FGF19, fibroblast growth factor 19; TJ proteins, tight-junction proteins.

In addition to clinical studies on T2DM patients, a clinical trial which recruited 20 healthy Korean participants found that metformin treatment altered the abundances of *Clostridium*, *Escherichia*, *Intestinibacter*, and *Romboutsia*, and the relative abundances of metabolites changed including carbohydrate, fatty acid, and amino acid metabolism (95). In experimental animal models, treatment with metformin was revealed to increase SCFA production, to reduce circulation LPS, to inhibit intestinal proinflammatory signaling activities, which was in line with clinical studies (80, 96, 97) (**Figure 2**). The activation of SCFA receptors, GPR41 and GPR43, stimulated the secretion of PYY and GLP-1, inhibiting appetite and improving insulin secretion. At the same time, increased-circulation SCFAs are responsible for improving energy metabolism, suppressing fat accumulation and insulin signaling in adipose tissue, and regulating the intestinal immunity and systemic inflammation (38, 39, 98). Accompanied by decreased LPS produced in the gut, metformin intervention increased goblet cell mass, mucin production, and tight-junction (ZO1 and occludin) proteins in obese gut, thereby relieving intestinal inflammation, decreasing leaky gut, and repairing the intestinal barrier structure (80, 96). In addition, the metabolic benefits of metformin might also be mediated by gut microbiota and bile acid homeostasis (76). Evidence shows that *Bacteroides fragilis* was decreased in samples from newly diagnosed T2DM patients after metformin treatment for 3 days, meanwhile the BA pool was altered (76). Bile acid glycoursodeoxycholic acid was increased, accompanied by inhibition of intestinal FXR signaling and decreased serum FGF19 levels (76). Reduced circulating FGF19 was found in subjects with metabolic disorders and hepatic steatosis, and FGF19 analogues have been identified as promising therapeutic methods in metabolic improvement (60). However, research associated with FGF19 was inconsistent, and the underlying mechanism still needs further research. Among the numerous gut flora altered during the metformin treatment in both clinical and experimental studies, *Akkermansia muciniphila*, a mucin-degrading bacterium, is related to healthy intestinal mucosa (79, 84, 96, 99). Furthermore, oral administration of *Akkermansia muciniphila* to high-fat diet-induced mice without metformin treatment significantly improved glucose homeostasis and reduced visceral adipose tissue inflammation by inducing Tregs, indicating the promising treatment value of *Akkermansia* spp. for T2DM (99).

In brief, in addition to activation of the master cellular energy sensor AMPK (74), metformin might act partly through gut microbiota and its metabolites to improve metabolic health. Notably, the metformin concentration in the gastrointestinal lumen is 30–300 times higher than in the circulation (100). High concentrations of metformin in the gastrointestinal lumen can increase glucose uptake and inhibit mitochondrial oxidative phosphorylation in enterocytes then accelerate glucose utilization through glycolysis and overproduction of lactate, the reason why metformin might contribute to gastrointestinal intolerance in a minority of people (71, 101, 102). Previous studies also hint that overproduction of lactate might also be microbially mediated (71, 103). Therefore, the potential mechanisms and contradiction of gastrointestinal intolerance and gut microbiota-related benefits need further investigation.

### SGLT2 Inhibitors

Sodium-glucose cotransporter 2 (SGLT2) inhibitors improve glycemic control by increasing renal glucose excretion, accompanied by pleiotropic non-glycemic properties, such as reductions in body weight and cardiovascular and renal protection effects (104–107). However, the underlying mechanism of the pleiotropic benefits was still not clear. Evidence shows that the protective effect might be explained for increased ketone body production in CVD, a clear fuel to improve the cardiac function of the energy-starved myocardium (108). As an orally ingested antidiabetic agent, experimental animal studies have found that SGLT2 inhibitor intervention slightly altered the microbiota composition in experimental animal studies (109–111) (summarized in **Table 2**).

**Table 2:** Experimental animal studies analyzing the effects of SGLT2 inhibitors on gut microbiota.

Anti-diabetic drugs	Animal model	Dose	Duration	Key results	Mechanism of action
Dapagliflozin (109)	C57BLKS/J-lepr^db^/lepr^db^	60 mg/kg diet	8 weeks	Actinobacteria, Bacteroidetes, Firmicutes, Proteobacteria and Verrucomicrobia altered *Oscillospira*, Firmicutes/Bacteroidetes ratios↓	Vascular function improvements effects not conclusively mediated by gut microbiota
Dapagliflozin (111)	Butyrate-supplemented db/db mice	1 mg/kg/day	6 weeks	*Streptococcus* spp.↑ *Adlercreutzia* spp. and *Alistipes* spp., Firmicutes/Bacteroidetes ratios↓	No big difference in the microbiota composition with Dapagliflozin intervention
Dapagliflozin (112)	STZ-induced HFD-fed Sprague Dawley rats	1 mg/kg/day	4 weeks	no effects on beneficial bacteria Proteobacteria (especially *Desulfovibrionaceae*)↑	No effects on beneficial bacteria
Dapagliflozin (110)	MafA-deficient mice	1 mg/kg/day	6 weeks	*Blautia*↑ *Clostridium perfringens, enterococci, Enterobacteriaceae*, and *intestinal enterococci*↓ Intestinal SCFAs↑	Regulated the intestinal microecological balance of the body and promoted blood glucose and energy homeostasis.
Canagliflozin (113)	CE-2 diet-induced mice	10 mg/kg/day	2 weeks	Actinobacteria, *Oscillospira*↓ Cecal SCFAs↑	Increased bacterial carbohydrate fermentation; Reduced the accumulation of uremic toxins including p-cresyl sulfate

STZ, streptozocin; HFD, high-fat diet; SCFA, short-chain fatty acids.

Dapagliflozin treatment showed minor beneficial alterations of gut microbiota in T2DM mice, a trend for decreased *Oscillospira* spp. and Firmicutes/Bacteroidetes ratios and increased *Akkermansia muciniphila* in the treatment group (109). In the butyrate-supplemented diet-fed db/db mice, the dapagliflozin-treated mice were also characterized by a decreased trend in Firmicutes/Bacteroidetes ratios, as well as a decreased trend in *Adlercreutzia* spp. and *Alistipes* spp. and an increased trend in *Streptococcus* spp (111).. In addition to slight alterations in gut microbiota, SGLT2 inhibitor intervention significantly improved intestinal SCFA production in animal models (110, 113). However, the results were inconsistent, and dapagliflozin treatment was found to have no beneficial effects on gut bacteria in diabetic rats (112). Only two clinical studies explored the alteration of fecal microbiome with SGLT2 inhibitor treatment (87, 88). Seventy-six treatment-naive T2DM with risk factors for CVD were included in a randomized, open-label, two-arm clinical trial (88). After a 3-month intervention, empagliflozin improved glucose metabolism and reduced CVD-related risks, while it significantly altered the gut microbiota, including an increase in SCFA-producing bacteria and a reduction in several harmful bacteria such as *Escherichia–Shigella*, *Bilophila*, and *Hungatella* (88). However, another clinical study found no significant effect on microbial alpha diversity or composition (87). It might be due to the fact that all of the subjects included had already been treated with metformin, which might have overshadowed the possible impact of dapagliflozin on the gut microbiome (87). Experimental studies found that dapagliflozin increased the abundance of *Desulfovibrionaceae*, which was increased in the fecal microbiota of animal models with metabolic disorders (114, 115), while metformin reduced *Desulfovibrionaceae*, suggesting that the combination drug therapy of dapagliflozin and metformin might have complementary actions on the gut microbiota in diabetes (112). Given all this, the pleiotropic beneficial effects of the SGLT2 inhibitor might be slightly mediated by gut microbiota or not be mediated by gut microbiota, and the potential mechanism of the pleiotropic beneficial effects of SGLT2 inhibitors need to be further uncovered (116).

### Thiazolidinedione Insulin Sensitizers

Thiazolidinedione (TZD) drugs are effective oral agents for T2DM in improving insulin sensitivity (117). TZDs are ligands of peroxisome proliferator-activated receptor gamma (PPAR-γ), leading to the activation of various pathways related to glycemic homeostasis and lipid metabolism (117, 118). The expression of PPAR-γ is abundant in the intestinal tract; thus, it is possible that PPAR-γ agonists straightly impact on gut microbiome homeostasis to improve energy metabolism (119, 120). However, only a few experimental animal studies explored whether TZD treatment can modify gut microbiota homeostasis (119, 121, 122). In a high-fructose-fed mouse model, pioglitazone partly altered gut microbiota and relieved the intestinal inflammation and epithelial barrier impairment, such as preventing the increment of the pathogenic bacteria *Deferribacteraceae* (*Mucispirillum*) (121). In diabetic mice, treatment with rosiglitazone promoted insulin sensitivity without modifying the composition of gut flora but improved the gene expression related to lipid and carbohydrate metabolism as well as immune regulation in the ileum and colon (119). Another experimental study discovered that microbial metabolites, for example, hippurate and indole-3-ethanol, were decreased by pioglitazone intervention in iNOS knockout mice (122). These experiment research suggested that TZDs might have mild protective effects on gut microbiota, mainly focused on lipid and carbohydrate metabolism and inflammation. However, no clinical study focused on gut microbiota and microbial metabolites alterations with TZDs treatment in T2DM subjects; further research is still needed.

### Dipeptidyl Peptidase-4 Inhibitors

Dipeptidyl peptidase-4 (DPP-4) inhibitors inhibit the degradation of glucagon-like peptide-1 (GLP-1) and glucose-dependent insulinotropic polypeptide to stimulate insulin secretion, reserve β-cell function, and maintain glucose homeostasis (123). A series of experimental studies have shown that DPP-4 inhibitors might be able to improve energy metabolism through shaping the gut microbial composition and increasing fecal SCFAs (124–127) (summarized in **Table 3**). In high-fat diet-induced obesity mice, DPP-4 inhibitors exerted an important impact on gut microbial composition and fecal metabolites, particularly the increased abundance of Bacteroidetes (124). Researchers then transplanted the fecal microbiota of DPP-4 inhibitor-treated patients to germ-free mice and observed an improved glucose intolerance (124). Compared with that in GLP-1 receptor agonist liraglutide-treated mice, the gut microbiota differed substantially in mice treated with DPP-4 inhibitors, indicating that the hypoglycemic mechanism of DPP-4 inhibitors on gut microbiota is at least not primarily by GLP-1 and the other potential benefit of DPP-4 inhibitors needs further research (124, 128). In addition to increment of SCFA-producing flora, DPP-4 inhibitors were found to reduce Toll-like receptor ligands and improve the production of antimicrobial peptides, exerting immunomodulatory and anti-inflammatory effects and maintaining intestinal homeostasis in obese mice, as well as cross talk with the liver and the whole-host health (126, 127). Some studies exhibited a decreased trend in the Firmicutes/Bacteroidetes ratio with treatment of DPP-4 inhibitors (124, 125, 127), while one experimental animal study found an enlarged abundance of Firmicutes and increased ratios of Firmicutes/Bacteroidetes (94). Although the relation between metabolic disorders and the Firmicutes/Bacteroidetes ratio is currently contradictory, more literatures considered it as a characteristic of obesity and T2DM (55).

**Table 3:** Experimental animal studies analyzing the effects of DPP-4 inhibitors on gut microbiota.

Anti diabetic drugs	Animal model	Dose	Duration	Key results	Mechanism of action
DPP-4 inhibitor (124)	HFD-fed C57BL/6	300 mg/kg/day of saxagliptin or 4 g/kg of sitagliptin	4 weeks	The changes of 68.6% genera induced by HFD were rescued by the DPP-4 inhibitor. Bacteroidetes↑ Firmicutes↓ Bacteroidales S24–7 group, Bacteroidaceae, Ruminococcaceae, Desulfovibrionaceae and Streptococcaceae↓ Fecal SCFAs (especially succinate) ↑	Increasing the production of succinate contributed to the hypoglycemic effect of DPP-4 inhibitor
DPP-4 inhibitors (125)	HFD-fed C57BL/6	15 mg/kg/day	12 weeks	Firmicutes/Bacteroidetes ratios↓ Ruminococcus, Dorea, Verrucomicrobia↑ Plasma sphingomyelin, phosphatidylcholine and lysophosphatidylcholine entities↓	Elevated levels of butyrate-producing flora Reduced levels of certain plasma sphingomyelin, phosphatidylcholine and lysophosphatidylcholine entities
Vildagliptin (127)	WD-fed C57BL/6	50 mg/kg/day	8 weeks	*Oscillibacter* spp., Ruminococcaceae↓ *Lactobacillus* spp.↑ Cecal propionate↑ Cecal TLR ligands↓	Promoted antimicrobial peptide production and increased crypt depth in the ileum Indirectly reduced the expression of proinflammatory cytokines in the liver
Sitagliptin (94)	Zucker diabetic fatty rats	10.76 mg/kg/day	4 weeks	*Lactobacillus* spp.↑Firmicutes↑ Firmicutes to Bacteroidetes ratios↑	Selectively increased the beneficial flora
Saxagliptin (128)	STZ-induced ApoE-/- C57BL/6 mice	80 mg/kg/day	8 weeks	No significant effect on microbial composition	No significant effect on microbial composition
Linagliptin (126)	HFRU-fed C57BL/6 mice	15 mg/kg/day	5 weeks	*Bacteroidetes* spp.↑ *Proteobacteria* spp.↓ *Zo-1* mRNA, *Mucin* mRNA↑	Attenuated hepatic steatosis by gut-liver axis modulation
Vildagliptin (129)	STZ-induced diabetic Sprague-Dawley rats	20 mg/kg/day	12 weeks	Firmicutes/Bacteroidetes ratios↓ *Baceroides* and *Erysipelotrichaeae*↑	Increased SCFAs production
Sitagliptin (130)	HF/HC-STZ Sprague-Dawley rat	10 mg/kg/day	12 weeks	Firmicutes↓ Bacteroidetes, Tenericutes↑	Increased SCFAs-producing bacteria and probiotic

STZ, streptozocin; HFD, high-fat diet; WD, Western diet; HFRU, high-fructose diet; HF/HC, high fat or high carbohydrate; SCFA, short-chain fatty acids; TLR, Toll-like receptors.

There existed a few clinical studies that explored the gut flora modifying the effect of DPP-4 inhibitors (89–91). However, in a clinical study which included 51 T2DM patients, the advantageous effect of sitagliptin on glucose control, weight loss, and BA metabolism was not related to alterations in the gut microbiota (89, 90). No significant effect on microbial composition was found, which is possibly due to the fact that these subjects previously used metformin or sulphonylureas as hypoglycemic therapies, and it might have covered the possible effects of DPP-4 inhibitors (89, 124). Another clinical study which included 90 T2DM subjects found that both vildagliptin and saxagliptin altered the composition of gut microbiota, respectively (91). Thus, the microbiota-shaping effects of DPP-4 inhibitors in clinical studies and its additional hypoglycemic mechanism need further investigation.

### α-Glucosidase Inhibitors

α-Glucosidase inhibitors are antidiabetic drugs, including acarbose, miglitol, and voglibose, which delay the absorption of carbohydrates in the intestinal tract to inhibit the rise in postprandial plasma glucose concentration (131). α-Glucosidase inhibitors are inhibitors of both human and bacterial α-glucosidases, and because of its high intestinal drug concentration, α-glucosidase usually has noticeable impacts on the intestinal flora (132, 133). Large amounts of research revealed that α-glucosidase inhibitors could shape the composition of the gut microbiome in both animal studies and clinical studies (62, 92–94, 134). Evidence shows that acarbose modulated the gut microbiota and corresponding shaped fecal and plasma BA composition, which may improve host energy metabolism (62, 135). A clinical study which recruited 51 treatment-naive T2DM patients showed that a three-mouth treatment with acarbose increased *Lactobacillus* and *Bifidobacterium* abundances and reduced *Bacteroides* abundances, along with altered plasm BA pool composition (62). Another clinical study which included 95 T2DM patients found that acarbose treatment improved the abundance of *Enterococcus faecalis* and *Bifidobacterium longum*, along with the reduction of plasma inflammatory factors, such as prothrombin activator inhibitor-1 and LPS levels (93). As summarized in **Table 4**, intervention with α-glucosidase inhibitors in experimental animal studies also confirmed significant impacts on gut microbiota and relevant metabolites. In addition to their glucose-lowering and energy metabolism-improving effects, α-glucosidase inhibitors were found to reverse joint inflammation on collagen-induced arthritis mice and the underlying mechanism might be due to the alteration of host–commensal interactions, which have been confirmed to be correlated with rheumatoid arthritis, such as several butyrate-producing species, *Lactobacillus* spp. and *Oscillospira* spp (48, 138, 141).. These results suggested a promising prospective of α-glucosidase inhibitors due to its potential antiarthritis effect mediated by the gut microbiome (134, 138).

**Table 4:** Experimental animal studies analyzing the effects of α-glucosidase inhibitors on gut microbiota.

Anti-diabetic drugs	Animal model	Dose	Duration	Key results	Mechanism of action
Acarbose (94)	Zucker diabetic fatty rats	32.27 mg/kg/day	4 weeks	Actinobacteria↑ *Bifidobacterium, Ruminococcus 2, Lactobacillus intestinalis*↑ Metagenomic functional prediction: elevated carbohydrate transport and metabolism.	Selectively increased the beneficial flora
Acarbose (134)	Old mice	1,000 ppm	8 months	*Muribaculaceae*↑ SCFA↑	Modulated the fermentation products of the gut flora
Acarbose (136)	HS or PP-fed mice	400 ppm	28 days	Diet-dependent gut community structure alteration and SCFA increasing	Increased SCFA production
Acarbose (137)	STZ-induced HFHSD-fed SD rats	30 mg/kg/day	7 weeks	*Escherichia-Shigella*↓ *Muribaculaceae, Lachnospiraceae, Bifidobacterium, Ruminococcaceae_UCG-014, Ruminococcus_1, Romboutsia, Eggerthellaceae, Alistipes, Faecalibaculum, Ruminococcaceae_UCG-013* and *Peptococcaceae*↑	Beneficial composition of gut microbiota restored
Acarbose or miglitol (138)	Collagen-induced arthritis mice	500 mg/kg/day	55 days	Firmicutes↑*Oscillospira* spp., *Desulfovibrio* spp. and *Ruminococcus* spp.↑ *Lactobacillus* spp., *Anaeroplasman* spp., *Adlercreutzia* spp., and *RF39* spp.↓	Regulated immunity *via* Th17/Treg cells in the intestinal lamina propria
Voglibose (135)	HFD-fed C57BL/6 mice	1 mg/kg/day	12 weeks	the ratio of Firmicutes to Bacteroidetes↓ Plasm taurocholic, cholic acid and deoxycholic acid↑	Downregulated gene expression of CYP8B1 and HNF4α Upregulated gene expression of PGC1α
Miglitol (139)	HFHSD-fed rats	0.04% miglitol plus in diet	12 weeks	*Erysipelotrichaceae* and *Coriobacteriaceae*↓ Plasm LPS↓	Reduced LPS levels in portal plasma
Miglitol (140)	ChREBP-knockout mice	0.08% miglitol plus in diet	8 weeks	Lactobacillales and *Bifidobacterium*↑ clostridium cluster XIVa↓ Fecal lactate↑	Increased cecal lactate contents and altered intestinal flora

STZ, streptozocin; HFD, high-fat diet; HS, high-starch; PP, plant polysaccharides; HFHSD, high-fat, high-sucrose diet; SCFAs, short-chain fatty acids; HNF4α, hepatocyte nuclear factor 4alpha; PGC1α, peroxisome proliferator-activated receptor-γ co-activator-1α; LPS, lipopolysaccharide.

In fact, over 95% of the acarbose dose was not absorbed in the gut, coupled with its feature to inhibit microbial α-glucosidases, and subjects’ treatment response to acarbose is dependent on several factors, such as dietary intake, genetic factor, and microbiota composition before treatment (also named enterotypes) (62, 91–93, 136, 142, 143). The acarbose-shaped gut microbial composition might be related to the dietary intake in a small Japanese population with T2DM (92). Moreover, hierarchical clustering showed that the habitual dietary intake of sucrose, fat, and carbohydrate was associated with three distinct microbial clusters, and even the abundance alteration of *Faecalibacterium* was positively related to dietary rice intake but negatively related to bread intake (92). A previous study also found that patients with a gut flora driven by *Bacteroides* displayed more beneficial modifications in gut microbiota, plasma BA composition, and more metabolic metabolism enhancement after acarbose treatment than those with *Prevotella* (62). In addition, researchers revealed that acarbose resistance has spread in certain host gut microbiomes, which contributed an emerging layer to the multifaceted network of carbohydrate-mediated cross talk among various human microbiomes (132, 144). Besides, in antibiotic pretreatment mice, whose gut microbial enzyme activities have been weakened, the metabolism of voglibose was reduced and more significantly glucose-lowering effects were presented (143). In brief, from currently clinical and experimental studies, α-glucosidase inhibitors have obvious effects on gut microbiota and its effects significantly depend on host diet and the original composition of the gut microbiome.

### Other Oral Glucose-Lowering Agents

Other less researched oral antidiabetes medications, such as sulfonylurea and glinide insulin secretagogues, have been noticed to cross talk with probiotic bacteria or microbial metabolic profiles (145–147). Nevertheless, two clinical studies which were designed to assess the effects of sulfonylureas on gut microbiota in T2DM subjects found no beneficial impacts on gut microbiota composition even in treatment-naive subjects, but with enhanced glycemic control (62, 87). At the same time, acarbose showed beneficial effects on the composition of the gut microbiome, suggesting that the detected metabolic modifications of sulfonylureas might not be intermediated by their impacts on the gut microbiota (62, 87). Recently, a few newly invented oral anti-glucose agents were discovered and used in clinical application, such as chiglitazar and imeglimin (148, 149). Activating as a peroxisome proliferator-activated receptor pan-agonist for glucose control, chiglitazar was found to improve insulin sensitivity and lipid homeostasis and reduce circulating levels of inflammatory parameters (150, 151). Imeglimin was confirmed to have the effects of modulating mitochondrial bioenergetics, enhancing mitochondrial function, improving insulin sensitivity, and preserving β-cell function (152–154). However, the associations of these anti-glucose agents and gut microbiota composition were still lacking.

In addition, newly identified exciting targets, including glucokinase activators and G-protein-coupled receptor 40 agonists, have also been researched, although not clinically usable (155, 156). Therefore, with the development of novel glucose-lowering agents, further research is still needed to uncover the complex interaction among gut microbiota, glucose-lowering agents, and the microbial-host metabolic cross talk.

## Conclusions and Future Prospects

Antidiabetic agents modify the gut flora and thereby alter gastrointestinal and plasma metabolite profiles, further improving metabolic health. Knowledge and studies so far indicate that oral antidiabetes drugs, including metformin, DPP-4 inhibitors, and α-glucosidase inhibitors, have obvious effects on gut microbiota and microbial metabolites, while SGLT2 inhibitors and TZDs have slighter effects (62, 77, 87, 89). Even if the definite microbial signatures linked to certain antidiabetic agents have not been discovered yet, understanding how antidiabetes drugs influence the gut microbiome might be vital for identifying their potential mechanisms and optimizing their treatment. Although different hypoglycemic drugs shape gut microbiota differently, they have been confirmed to have some similar effects in regulating microbiota and metabolites. Among various microbiota and metabolites derived from gut flora, metformin, SGLT2 inhibitors, DPP-4 inhibitors, and α-glucosidase inhibitors have been demonstrated to have similar effects on increased SCFA-producing bacteria and SCFA production, which may partly explain their beneficial effects in the regulation of insulin sensitivity enhancement, energy metabolism, and systemic inflammation (77, 78, 110, 124, 134). Notably, among various SCFA-producing bacteria, *Akkermansia muciniphila* has been proven increased particularly during the metformin treatment in both clinical and experimental studies, which also related to healthy intestinal mucosa and anti-inflammatory action (79, 84, 96, 99). In addition, alteration of the BA pool was commonly displayed in both metformin and α-glucosidase inhibitors, corresponding with decreased *Bacteroides fragilis* in metformin-treated individuals and increased *Lactobacillus* and *Bifidobacterium* abundances and reduced *Bacteroides* abundances in α-glucosidase inhibitor-treated individuals, respectively (62, 76). In addition, reduction of opportunistic pathogen and attenuated intestinal inflammation could be seen in intervention research on metformin, DPP-4 inhibitors, and α-glucosidase inhibitors (77, 126, 138). Therefore, manipulation of gut microflora composition could be a potential and promising target to improve metabolic outcomes in subjects with T2DM. The microbiota–host cross talk might convey novel and potential ideas of generally used oral glucose-lowering drugs.

Firstly, combination therapy might have additional benefits, due to the fact that different antidiabetes drugs shape gut microbiota with distinct effects (62, 94, 112), For example, dapagliflozin increased the abundance of *Desulfovibrionaceae* in a T2DM rat model, which is an unfriendly sulfate-reducing bacteria in the gut, while metformin reduced it on the contrary, revealing a rationality and complementary action of combined pharmacotherapy between dapagliflozin and metformin (112). However, the definite combination effects of metformin and SGLT2 inhibitors need further investigation. T2DM is a chronic disease with progressive features and possible complex complications; a satisfactory treatment effect is hard to achieve with monotherapy. Besides metformin and SGLT2 inhibitor combined treatment, combination therapies, such as metformin with pioglitazone or metformin with DPP-4 inhibitors, might exhibit a synergetic role in gut microbiome benefits (157, 158). Further investigations in both experimental and clinical are needed to figure out the combined pharmacotherapy effects on gut microbiota.

Secondly, pre- and probiotics could be a promising treatment for T2DM in the modulation of gut microbiota (159). For example, *Actinoplanes* spp. and *Lactobacillus* spp. have been definitively demonstrated to effectively inhibit the alpha-glucosidase activity to reduce glucose levels (160, 161). The combination of hypoglycemic agents and certain probiotics or prebiotics may further enhance the glucose-lowering effects (82, 162). Prebiotics, such as inulin and galacto-oligosaccharide, could be fermented by the gut flora, leading to modulation of intestinal microbiota and the production of various microbial metabolites including SCFAs (163–165). Besides, evidence shows that combination of metformin and gastrointestinal microbiome modulator (consisting of inulin, beta-glucan, and polyphenols) treatment significantly relieved metformin tolerance than the placebo combination (166). Notably, for patients with pregestational diabetes and gestational diabetes mellitus (GDM), the dominating pharmacotherapy is insulin, while only metformin and glyburide are used in some countries (167, 168). Other oral hypoglycemic agents are limited in these patients. Hyperglycemia during pregnancy is associated with significantly increased maternal and fetal metabolic disturbance and morbidity (167). Therefore, dietary modification and physical activity are particularly important for glycemia control (167). A systematic review and meta-analysis revealed that probiotic supplementation in GDM could significantly reduce homeostasis model assessment of the insulin resistance index with no adverse effects reported (169). Evidence shows that inulin-type fructan supplementary improved glucose and lipid metabolism in HFD-induced GDM mouse models associated with gut flora modification (170). Our research team found that maternal inulin treatment improved glucose metabolism in adult male offspring *via* regulation of the hepatic long non-coding RNA profile (164). However, results are inconsistent showing that probiotics, including *Lactobacillus rhamnosus* and *Bifidobacterium animalis* subspecies *lactis*, did not prevent GDM in overweight and obese pregnant women (171). Thus, more clinical studies are needed to verify these results and explore the ideal bacterial composition of pre- and probiotics that might positively alter glucose metabolism in GDM or pregestational diabetes.

Thirdly, FMT from normal glucose tolerance or antidiabetes treatment subjects to mice revealed a significant improvement in gut microbiota composition, glucose homeostasis, and metabolic health (17, 124, 172). Despite that this promising treatment was still in its infancy (173–175), FMT combined with antidiabetes drugs might bring novel interventions and perspectives in T2DM management. The effects and mechanisms underneath these potential treatment schedules are still unclear, and it is vital to further develop meaningful and applicable interventions combined with intestinal microbiota in the future study.

## Author Contributions

DW: writing—original draft preparation. JL, LZ, QZ, ML, and XX: writing—review and editing. XX: supervision. XX and QZ: funding acquisition. All authors contributed to the article and approved the submitted version.

## Funding

This work was supported by the grants from the National Natural Science Foundation of China (No. 82170854, 81870579, 81870545, 81570715, 81170736), Beijing Natural Science Foundation (7202163), Beijing Municipal Science & Technology Commission (Z201100005520011), CAMS Innovation Fund for Medical Sciences (CIFMS2021-1-I2M-002).

## Conflict of Interest

The authors declare that the research was conducted in the absence of any commercial or financial relationships that could be construed as a potential conflict of interest.

## Publisher’s Note

All claims expressed in this article are solely those of the authors and do not necessarily represent those of their affiliated organizations, or those of the publisher, the editors and the reviewers. Any product that may be evaluated in this article, or claim that may be made by its manufacturer, is not guaranteed or endorsed by the publisher.
